# Evidence of cellular stress and caspase-3 resulting from a combined two-frequency signal in the cerebrum and cerebellum of Sprague-dawley rats

**DOI:** 10.18632/oncotarget.11753

**Published:** 2016-08-31

**Authors:** Alberto López-Furelos, José Manuel Leiro-Vidal, Aarón Ángel Salas-Sánchez, Francisco José Ares-Pena, María Elena López-Martín

**Affiliations:** ^1^ Department of Morphological Sciences, Faculty of Medicine, University of Santiago de Compostela, Santiago de Compostela, Spain; ^2^ Institute of Alimentary Analysis, University of Santiago de Compostela, Santiago de Compostela, Spain; ^3^ Department of Applied Physics, Faculty of Physics, University of Santiago de Compostela, Santiago de Compostela, Spain

**Keywords:** heat shock proteins, specific absorption rate, caspase-3, multiple electromagnetic fields, cerebral cortex, Pathology Section

## Abstract

Multiple simultaneous exposures to electromagnetic signals induced adjustments in mammal nervous systems. In this study, we investigated the non-thermal SAR (Specific Absorption Rate) in the cerebral or cerebellar hemispheres of rats exposed in vivo to combined electromagnetic field (EMF) signals at 900 and 2450 MHz.

Forty rats divided into four groups of 10 were individually exposed or not exposed to radiation in a GTEM chamber for one or two hours. After radiation, we used the Chemiluminescent Enzyme-Linked Immunosorbent Assay (ChELISA) technique to measure cellular stress levels, indicated by the presence of heat shock proteins (HSP) 90 and 70, as well as caspase-3-dependent pre-apoptotic activity in left and right cerebral and cerebellar hemispheres of Sprague Dawley rats.

Twenty-four hours after exposure to combined or single radiation, significant differences were evident in HSP 90 and 70 but not in caspase 3 levels between the hemispheres of the cerebral cortex at high SAR levels. In the cerebellar hemispheres, groups exposed to a single radiofrequency (RF) and high SAR showed significant differences in HSP 90, 70 and caspase-3 levels compared to control animals. The absorbed energy and/or biological effects of combined signals were not additive, suggesting that multiple signals act on nervous tissue by a different mechanism.

## INTRODUCTION

Exposure of the general population to radiofrequency (RF) fields from mobile phones, wireless networks, television, radio broadcasting and other communication technologies has become universal and continuous in recent years. Humans are also simultaneously exposed to multi-signal RF and there is very little information about the biological effects of this. Only a very few epidemiological [1, 2], *in vitro* [3] or *in vivo* [4, 5] studies have been done for exposure to combined RF. It remains unclear whether there are repercussions on human health.

In spite of a general consensus that radiofrequency can affect EEG and other markers of cerebral function [6] in humans, there is insufficient data to indicate that the interaction of multiple RF affects the physiology of nervous tissue.

Electromagnetic fields (EMF) can act as inducers of cellular stress and provoke the synthesis of cytoprotective heat shock proteins (HSP) [7, 8]. Increased HSPs in tissue is often associated with a resistance to or decrease in apoptosis (programmed cellular death) [9]. HSP 70 is one of the main proteins induced by stress in the nervous system and its neuroprotective function has been demonstrated *in vivo* as well as *in vitro* [10]. Recent studies suggest that exposure to microwaves may induce apoptosis of the nervous cells, as increased caspase-3 indicates pre-apoptotic activation of the caspase-dependent mitochondrial pathway [11]. The biological effects described are related to the duration, frequency and intensity of the EMF [12]. However, there are no studies that provide information about stress-induced cellular change after simultaneous exposure to several frequencies, which is the most common scenario for EMF interaction in humans. We recently carried out a study with an experimental multi-frequency model on eight different rat tissues [13]. The absence of acute tissue effects in that work failed to answer: 1) whether absorbed energy and/or biological effects are additive in tissue, and 2) if the interaction mechanism for combined frequencies is the same as that for a single frequency in live tissue.

To continue searching for answers, we exposed Sprague-Dawley rats to radiation in an experimental multi-frequency radiation system and calculated the combined SAR by FDTD. We then analyzed cellular stress effects by studying HSP 90 and 70 along with the effects of pre-apoptotic caspase-3 activity in left and right hemispheres of the cerebrum and cerebellum.

## RESULTS

### Signal simulation and SAR calculation

Each of the pure sinusoidal signals used in Group I (900 MHz) and II (2450 MHz), and the sum of both signals (900 + 2450 MHz) can be seen in Figure 2A, which illustrates the simulations using MATLAB scientific software. The combination of signals obtained in the simulation was also validated at lower frequencies in the laboratory using the Agilent Infinium (600 MHz) oscilloscope to visualize the output signal that results from the sum of the two sinusoidal signals.

**Figure 1:**
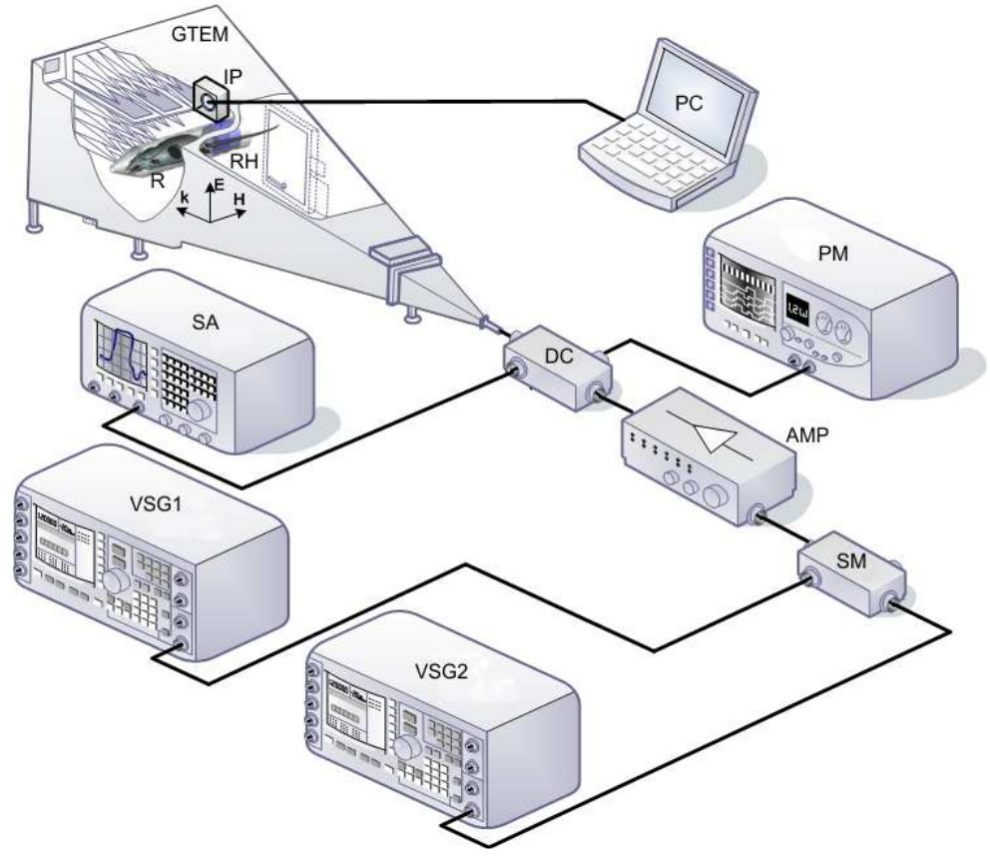
Schematic of the system GTEM, Schaffner 250 GTEM chamber; VSG1, Agilent E8267D vector signal generator (250 KHz-20 GHz) operating at 2.45 GHz; VSG2, Agilent E4438C vector signal generator (250 KHz-4 GHz) operating at 900 MHz; AMP, research amplifier 15S1G3 (0.8-3 GHz); DC, NARDA 3282B-30 directional coupler (800-4000 MHz); SA, Agilent E4407B spectrum analyzer (9 KHz-26.5 GHz); PM, Agilent E4418B power meter; SM, Agilent 11636a signal mixer; RH, rat holder; IP, EF Cube isotropic probe; R, rat.

**Figure 2:**
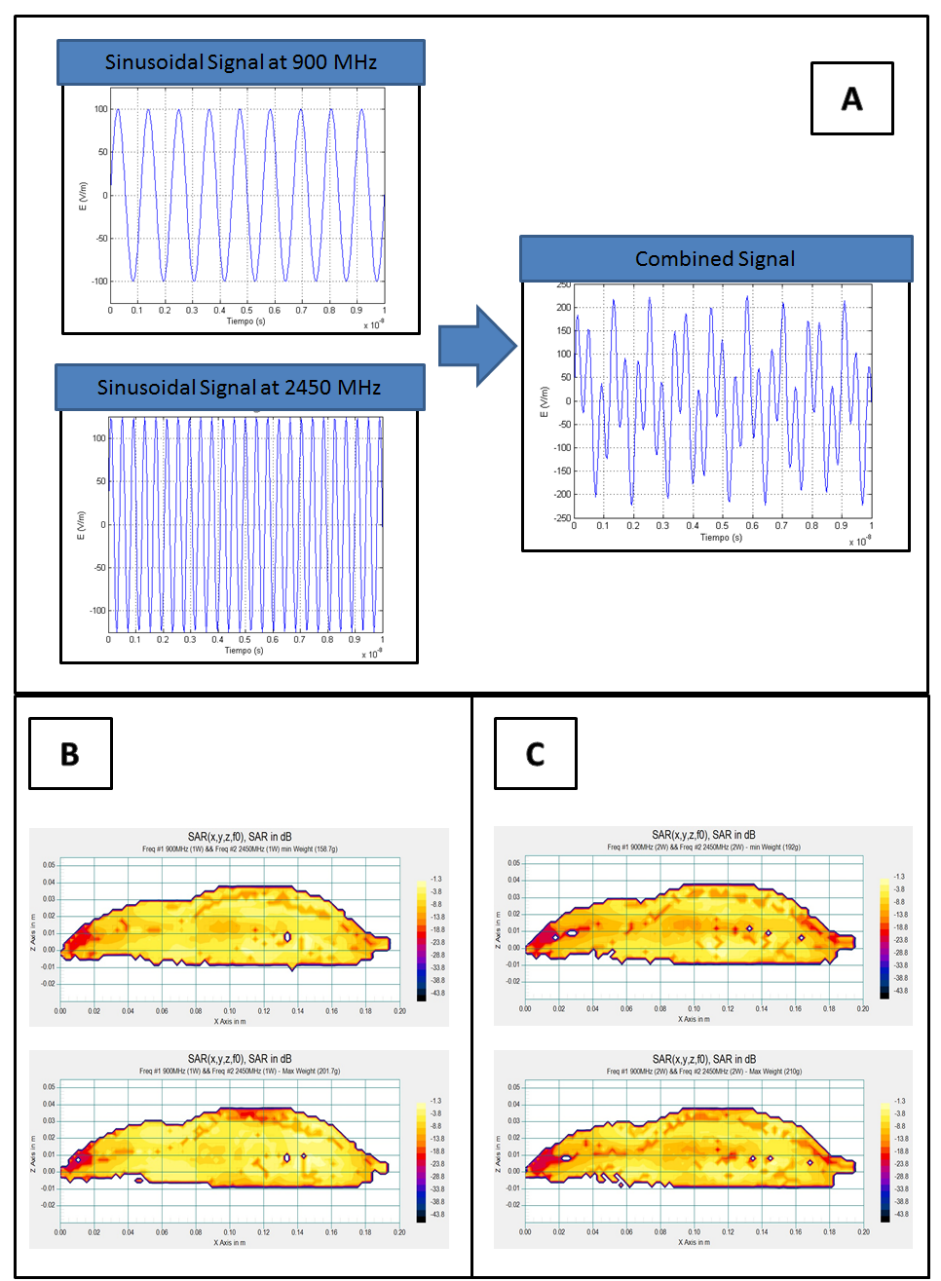
**A.** Representation of each of the pure sinusoidal signals used in Group I (900 MHz) and II (2450 MHz) and the sum of both signals (900 + 2450 MHz). **B.** Distribution of Mean SAR in vertical sections of the scaled numerical phantom rat when radiated simultaneously with 900 MHz (P_TR_ = 1 W) and 2450 MHz (P_TR_ = 1 W) for cases of minimum and maximum mass of the group. SAR is expressed relative to absorption of the entire local power density in the absence of the rat. **C.** Distribution of Mean SAR in vertical sections of the scaled numerical phantom rat when radiated simultaneously with 900MHz (P_TR_ = 2 W) and 2450 MHz (P_TR_ = 2 W) for cases of minimum and maximum mass of the group. SAR is expressed relative to absorption of the entire local power density in the absence of the rat.

Tables I and II show how the SAR_E_ values in radiated rat groups I to III (eq. (1)) in the cerebrum, cerebellum and body correspond to the mean SAR value for each tissue type. Figures 2B and 2C show the mean SAR distribution in vertical sections of the scaled numerical phantom rat when radiated with 900MHz (power = 1W or 2W) and 2450MHz (power = 1W or 2W) simultaneously.

**Tables I and II:** SAR values in cerebrum, cerebellum and body of experimental rats, calculated from transmitted power (P_TR_), electric field (E_m_) and incident power density (P_D_)

	Weight [g]	FDTD-calculated specific absorption rate: experimental measurements		
		Mean SAR of body [W/kg]	Mean SAR of cerebrum [W/kg]	Mean SAR of cerebellum [W/kg]
**GROUP I:** f=900MHz P_TR_=2W E_m_ =48V/m P_D_=6.11W/m^2^	185.9	0.0541	0.0583	0.0212
	198.4	0.0510	0.0513	0.0320
**GROUP II:** f=2450MHz P_TR_=2W E_m_ =50V/m P_D_=6.63W/m^2^	175.7	0.0723	0.2731	0.1590
	193.9	0.0682	0.2344	0.1094
**GROUP III:** f=900+2450MHz P_TR_=1W+1W E_m_ =39 V/m P_D_=4.03W/m^2^	158.7	0.0478	0.0212	0.0678
	201.7	0.0367	0.0183	0.0422

**Table:** 

	Weight [g]	FDTD-calculated specific absorption rate: experimental measurements		
		Mean SAR of body [W/kg]	Mean SAR of cerebrum [W/kg]	Mean SAR of cerebellum [W/kg]
**GROUP I:** f=900MHz P_TR_=4W E_m_=62 V/m P_D_=10.20W/m^2^	191.9	0.0870	0.0732	0.0452
	219.6	0.0829	0.0934	0.0822
**GROUP II:** f=2450MHz P_TR_=4W E_m_ =57 V/m P_D_=8.62W/m^2^	186.7	0.0919	0.3274	0.1765
	207.3	0.0838	0.2572	0.1224
**GROUP III:** f=900+2450MHz P_TR_ =2W+2W E_m_=53 V/m P_D_=7.45W/m^2^	192.0	0.0705	0.1590	0.0856
	210.0	0.0776	0.1622	0.0894

### Stress levels measured by changes in post-irradiation rectal temperature

The difference in mean values among interaction levels of one or more frequencies was statistically significant (*p = 0.029*) in animals exposed to irradiation at 2 watts power and greater than would be expected after allowing for the random effects of differences in temperature changes before and after radiation. There was no statistically significant (*p = 0.433*) difference in temperature before and after radiation in any exposed group. The multiple comparison procedures (Bonferroni *t*-test) showed significant differences (*p = 0.025*) after irradiation between Group III (multi-frequency exposure) and Group IV (non- irradiated). See Table III.

**Table III:** Mean rectal temperature ±SEM before and after radiation at 2 W power

	RECTAL TEMPERATURES IN RATS EXPOSED TO 2W	
Experimental groups	Before radiation	After radiation
**Group I**	37.560 ±18×10^−2^	36.698± 4×10^−1^
**Group II**	37.125± 15×10^−2^	37.012±16×10^−2^
**Group III**	37.427±9×10^−2^	37.493±2×10^−1^^*^
**Group IV**	37.070±19×10^−2^	36.935±10×10^−2^

* Statistically significant differences between groups exposed to one or more interacting frequencies. * Indicates significant differences after radiation compared to the non-radiated control (GIV).

The animals exposed to irradiation at 4 watts power did not present differences in mean values for the interaction of one or more frequencies that were statistically significant (*p* = *0.359*) or great enough to exclude the possibility of difference due to random sampling variability after allowing for the effects of changes in temperature before and after radiation.

The difference in the mean values for temperature changes before and after radiation was statistically significant (*p* = *0.011*) and greater than would be expected after allowing for random effects of differences in the interaction of one or more frequencies. The effect of one or more frequencies was not statistically significant (*p* = *0.132*) and did not depend on the level before or after radiation. There was no interaction between the two factors. Using multiple comparison procedures (Bonferroni *t*-test), we detected statistically significant differences before and after irradiation in the animal groups subjected to a single radio frequency (Groups I and II) (*p = 0.07)* or the interaction of two radiofrequencies (Group III) (*p = 0.012)*, but not in the non-irradiated animals (*p = 0.839*). See Table IV.

**Table IV:** Mean rectal temperature ±SEM before and after radiation at 4 W power

	RECTAL TEMPERATURES IN RATS EXPOSED TO 4W	
Experimental groups	Before radiation	After radiation
**Group I**	37.493±2.7× 10^−1^	36.580±3.9× 10^−1^^#^
**Group II**	37.267±2.3× 10^−1^	36.400±2× 10^−1^^*^^#^
**Group III**	37.610±1.5× 10^−1^	36.447±4× 10^−2^
**Group IV**	37.290±5.7× 10^−1^	36.923±5× 10^−1^

* Statistically significant differences between groups exposed to one or more interacting frequencies;

# Statistically significant differences before and after radiation.

* Indicates significant differences after radiation compared to the non-radiated control (GIV).

### ChELISA results

#### In the cerebral cortex

#### HSP-90

The difference in mean values at both levels in the hemispheres of the cerebral cortex after exposure to 2 watts (right/left) was statistically significant (*p = 0.011*) and greater than would be expected after allowing for random effects of differences from one or more RF. We used a multiple comparison procedure to isolate the differing group(s).

The difference in the mean values among RF was not statistically significant (*p = 0.38*) or great enough to exclude the possibility of difference due to random sampling variability after allowing for the effects of differences in the hemispheres of the cerebral cortex.

**Figure 3:**
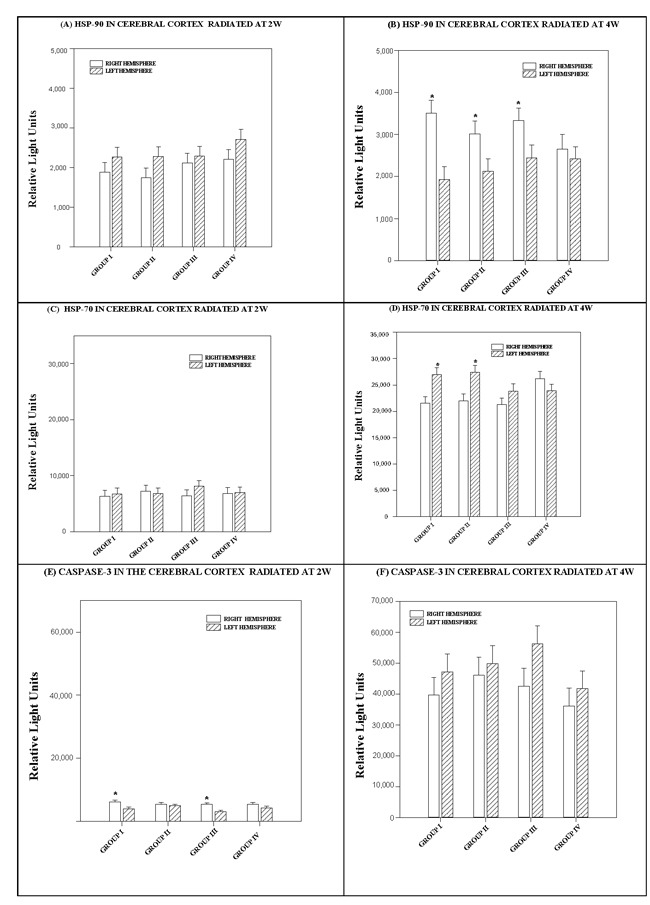
Histograms depicting the means and standard deviations of the chemiluminescence values for (A, B) HSP 90, (C, D) HSP 70 and (E, F) caspase-3 in rats radiated at 2W or 4W, in the right and left hemispheres of the cerebral cortex for each group: GI (900 MHz), GII (2.45 GHz), GIII (0.9+2.45 GHz) and GIV (control) * indicates significant differences (*p* < *0.05*) between right and left hemispheres.

The effects observed in each cerebrum hemisphere did not depend on the level or number of RF. There was no statistically significant interaction between cerebral cortex hemispheres and RF (*p = 0.75*) See Figure 3A.

The difference in the mean values at either level of each cerebral hemisphere at 4 watts power was statistically significant (*p < 0.001)* and greater than would be expected after allowing for random effects of differences from one or more RF. A multiple comparison procedure (Bonferroni *t*-test) was used to isolate the differing group(s). Significant differences in HSP-90 values were detected between the right and left cerebral hemispheres in Groups I (*p* < *0.001*), II (*p* = *0.041*) and III (*p = 0.042),* but not in *Group IV (p* = *0.625*). See Figure 3B.

The difference in mean values among RF was not statistically significant (*p = 0.663*) or great enough to exclude the possibility of difference due to random sampling variability after allowing for the effects of differences in cerebral hemispheres.

Effects in the cerebrum hemispheres did not depend on RF levels. There was no statistically significant interaction between cerebral cortex hemispheres and RF (*p* = 0.211).

#### HSP-70

The difference in mean values for each cerebral hemisphere after applying 2 watts power was not statistically significant (*p = 0.545*) or great enough to exclude the possibility of difference due to random sampling variability after allowing for the effects of differences in RF.

The difference in the mean RF values was not statistically significant *(p = 0.911*) or great enough to exclude the possibility of difference due to random sampling variability after allowing for the effects of differences in the cerebral hemispheres.

Effects at either level of each cerebral hemisphere did not depend on of the level of RF. There was no statistically significant interaction between them (*p = 0.771*) (see Figure 3).

The difference in the mean values for each hemisphere of the cerebral cortex exposed at 4 watts power was statistically significant (*p = 0.003*) and greater than would be expected after allowing for random effects of differences in RF. We used a multiple comparison procedure to isolate the differing group(s). HSP-70 values were significantly higher in the left hemisphere in Groups I (*p < 0.003*) and II (*p = 0.003*), but not in Groups III (*p = 0.162*) or IV *(p = 0.217)*.

The difference in the mean RF values at different levels was not statistically significant (*p = 0.239*) or great enough to exclude the possibility of difference due to random sampling variability after allowing for the effects of differences in the hemispheres of the cerebral cortex.

Effects at either level of each cerebral hemisphere depended on the RF level. There was a statistically significant interaction between cerebrum hemispheres and RF (*p = 0.010*).

#### Caspase-3

The difference in the mean values for each cerebral hemisphere exposed at 2 watts was statistically significant (*p < 0.001*) and greater than would be expected after allowing for random effects of the different radiofrequencies. We used a multiple comparison procedure to isolate the differing group(s). Significant differences were detected in the caspase-3 values of the right and left hemispheres in Groups I (*p < 0.006*) and III (*p = 0.003*), but not in Groups II *(p = 0.578)* and IV *(p = 0.140)* (see Figure 3E).

The difference in mean RF values at different levels was not statistically significant *(p = 0.320*) or great enough to exclude the possibility of difference due to random sampling variability after allowing for the effects of differences in both hemispheres.

Effects in each hemisphere did not depend on the RF level. There was no statistically significant interaction between hemispheres and radiofrequencies (*p = 0.306*).

The difference in the mean values for each cerebral hemisphere exposed at 4 watts was not statistically significant (*p = 0.063*) or great enough to exclude the possibility of difference due to random sampling variability after allowing for the effects of differences in radiofrequencies.

The difference in the mean values among the different RF levels was not statistically significant (*p = 0.259*) or great enough to exclude the possibility of difference due to random sampling variability after allowing for the effects of differences in both hemispheres.

The effect of different levels in either hemisphere did not depend on RF level. There was no statistically significant interaction between hemispheres and radiofrequencies (*p = 0.*837) (see Figure 3F).

#### In the cerebellum

#### HSP-90

The difference in mean values in the hemispheres of the cerebellum at 2 watts was not statistically significant (*p = 0.691*) or great enough to exclude the possibility of difference due to random sampling variability after allowing for the effects of differences in radiofrequencies.

The difference in mean values at different RF levels was not statistically significant (*p = 0.543*) or great enough to exclude the possibility of difference due to random sampling variability after allowing for the effects of differences between the cerebellar hemispheres.

**Figure 4:**
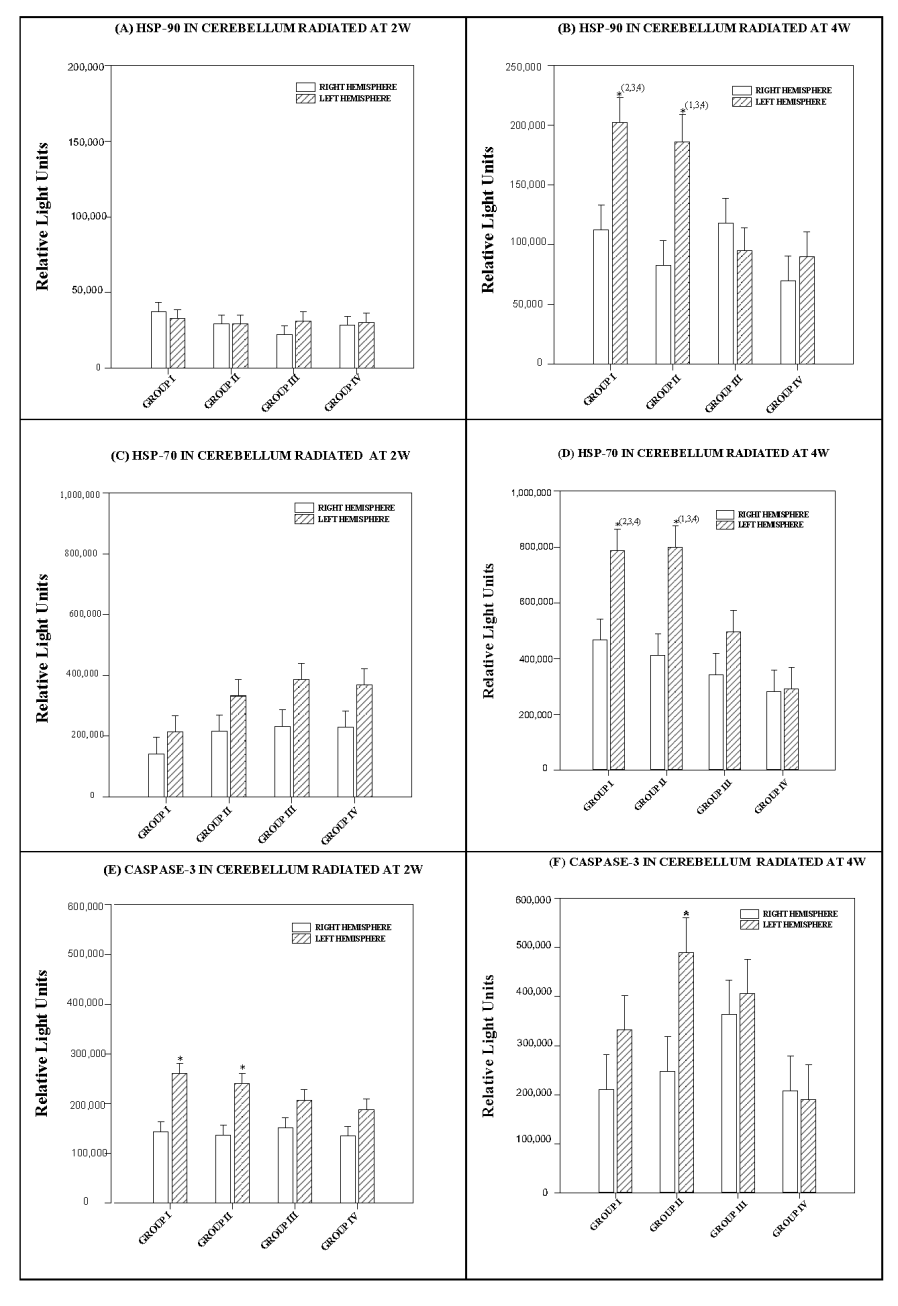
Histograms depicting the means and standard deviations of the chemiluminescence values for (A, B) HSP-90 (C, D) HSP-70 (E, F) caspase-3 in rats radiated at 2W or 4W, in the right and left hemispheres of the cerebellum for each group: GI (900 MHz), GII (2.45 GHz), GIII (0.9+2.45 GHz) and GIV (control) * indicates significant differences (*p* < *0.05*) between right and left hemispheres. ^(1,2,3,4)^ indicates significant differences (*p* < *0.05*) between groups.

Effects in the cerebellar hemispheres did not depend on the RF level. There was no statistically significant interaction between cerebellar hemispheres and radiofrequencies (*p = 0.713*) (see Figure 4A).

The difference in mean values for the hemispheres of the cerebellum at 4 watts was statistically significant (*p < 0.001*) and greater than would be expected after allowing for random effects of the different radiofrequencies. We used a multiple comparison procedure to isolate the differing group(s).

The HSP-90 level was significantly higher in the left hemisphere of the cerebellum in Group I (*p = 0.003)* and in Group II, which was subjected to two frequencies *(p < 0.001*).

The difference in the mean values for each radiofrequency was statistically significant (*p = 0.002*) and greater than would be expected after allowing for random effects due to differences in the cerebellar hemispheres of the animals of the animals. We used a multiple comparison procedure to isolate the differing group(s). The right cerebellar hemispheres of animals that received different types of radiation presented no significant differences in protein levels. However, significant differences in protein levels appeared in the left cerebellar hemisphere between Group I (900 MHz) and Groups III (900+2450 MHz) or IV (no radiation) (*p = 0.001* and *p = 0.005).* There were also differences between Group II and Groups III and IV (*p = 0.021* and *p = 0.05)* (see Figure 4B).

Effects in the cerebellar hemispheres depended on the RF level. There was a statistically significant interaction between cerebellar hemispheres and effects of the different radiofrequencies (*p = 0.012).*

#### HSP-70

The difference in mean values in the hemispheres of the cerebellum at 2 watts was statistically significant (*p = 0.009*) and greater than would be expected after allowing for random effects of different radiofrequencies. We used a multiple comparison procedure to isolate the differing group(s).

The difference in the mean values among the different RF was statistically significant (*p = 0.03*) and greater than would be expected after allowing for random effects due to differences in the cerebellar hemispheres. We used a multiple comparison procedure to isolate the differing group(s).

Effects in the cerebellar hemispheres at 2 watts power did not depend on the different RF levels. There was no statistically significant interaction between differences in the cerebellar hemispheres and the effects of the different radiofrequencies (*p = 0.984*) (see Figure 4C).

The difference in mean values in the hemispheres of the cerebellum at 4 watts was statistically significant (*p < 0.001*) and greater than would be expected after allowing for random effects of differences in radiofrequencies. We used a multiple comparison procedure to isolate the differing group(s). HSP-70 was significantly higher in the left hemisphere of the cerebellum in Group I (*p = 0.004*) and Group II (*p < 0.001*). See Figure 4D.

The difference in the mean RF values was statistically significant (*p* < *0.001*) and greater than would be expected after allowing for random effects due to differences in the cerebellar hemispheres at 4 watts power. We used a multiple comparison procedure to isolate the differing group(s). HSP-70 values from the left hemisphere of Groups I and II, which were subjected single frequencies of 900 and 2450 MHz, respectively, were significantly higher than those of animals exposed to two radiofrequencies (*p = 0.049* and *p = 0.035*) and non-radiated Group IV animals (*p < 0.001* in both cases). See Figure 4D.

Effects in the cerebellar hemispheres did not depend on differences in radiofrequencies. There was no statistically significant interaction between the cerebellar hemispheres and the different RF (*p = 0.064*).

#### Caspase-3

The difference in the mean values for the hemispheres of the cerebellum in animals exposed at 2 watts power was statistically significant (*p < 0.001*) and greater than would be expected after allowing for the random effects of different radiofrequencies. We used a multiple comparison procedure to isolate the differing group(s). Caspase-3 was significantly higher in the left cerebellar hemisphere of animals in Groups I and II (*p < 0.001* in both cases).

The difference in mean values at the different radiofrequencies in animals exposed at 2 watts power was not statistically significant (*p = 0.241*) or great enough to exclude the possibility of difference due to random sampling variability after allowing for the effects of differences in the cerebellar hemispheres.

Effects in the cerebellar hemispheres in animals exposed at 2 watts did not depend on differences in radiofrequencies. There was no statistically significant interaction between the cerebellar hemispheres and the different RF (*p = 0.271*) (see Figure 4E).

The difference in the mean values for the cerebellar hemispheres in animals exposed at 4 watts power was not statistically significant (*p = 0.054*) or great enough to exclude the possibility of difference due to random sampling variability after allowing for the effects of the different radiofrequencies.

Caspase-3 was significantly higher in the left cerebellar hemisphere of Group II animals (*p = 0.016)*, but not in the other groups.

The difference in mean RF values was statistically significant (*p = 0.030*) and greater than would be expected after allowing for random effects in the cerebellar hemispheres exposed at 4 watts. We used a multiple comparison procedure to isolate the differing group(s).

Caspase-3 values in the left hemisphere of Group II animals, which were subjected to 2450 MHz, were significantly higher than in non-radiated Group IV animals *(p = 0.018)* (see Figure 4F).

Effects in the cerebellar hemispheres exposed at 4 watts did not depend on differences in RF. There was no statistically significant interaction between the cerebellar hemispheres and the effects of the different RF. (*p = 0.290*).

## DISCUSSION

The apparent absence of biological effects on rat tissue morphology was inconclusive in a prior study [13] that compared biological effects of a single RF with those of two simultaneous RF signals. In contrast, the current work allowed us to experimentally determine that the biological effects of the simultaneous interaction of radiofrequencies were neither additive nor greater than the effects of applying each signal separately.

We have found no precedent for the results obtained in this work, in which exposure to one or two sources of radiation increased cellular heat stress (HSP) and pre-apoptotic (caspase-3) levels at sub-thermal SAR levels and different responses were observed between the right and left hemispheres of the cerebral cortex or cerebellum of rodents. Unlike other experimental models that look at the effects of combined RF exposure on neuronal cells [3], testicular tissue [4] or changes in tissue morphology, function or the cellular cycle with chronic exposure. [6], function or the cellular cycle were not evident. Though recent epidemiological studies have reported symptoms of cephalea and insomnia in different human population groups exposed to combined RF [1, 2], the cause is still being debated and it is not yet known whether the combined radiation exposure of various sources could have relevant health effects.

In this experimental work we observed radiation parameters that acted independently of the number of RF applied. Thus, in spite of the apparent attenuation of the biological effect of combined radiation with respect to single radiation, the increase in SAR > 0.33 W/kg provoked important increments in HSP levels, as has been described by other authors for the single frequency of 2.45GHz [8] at higher SAR (6W/kg). Time of exposure to the EMF may also play an important role in increasing HSPs [10, 8], even though stress levels measured by rectal temperature [14, 9] immediately after radiation decreased in the animals that were immobilized for two hours. The explanation is related to a decrease or compensation of the stress levels that counteract possible increases in animal temperature. The hypothalamus is the thermo-regulatory center in mammals and maintains homeostasis, adjusting body temperature [15, 16]. We are working with the following explanatory hypotheses: a) initial stress from immobilizing the animals provokes a sharp increase in temperature (in short periods such as 1 hour in Experiment 1) that will disappear during longer periods (2 hours in Experiment 2) as the animal becomes accustomed to immobilization; and b) radiation can provoke stimulation of the opiate receptors. There may be a balance between the potentially analgesic effect [17] of acute exposure to electromagnetic fields and the acute stimulus of immobilization (for one or two hours in this case) that generates stress and increases cortisol levels [18].

Another important finding was the significant differences in HSP levels that we observed between hemispheres in the cerebral cortex and cerebellum 24 hours after radiation at both SAR levels studied. The differences were more pronounced at the higher SAR. The cause of these differences might be related to small differences in EMF intensity (E), which had been previously tested experimentally on both sides of the animals' heads. This would explain the increased laterality of the protein response in the left hemisphere, which has also been observed in cerebral excitability of the hemispheres [19] or cellular proliferation [20] in humans exposed to radiation from mobile phones. Accordingly, the left side received higher EMF values than the right, which would have different repercussions in the cerebral cortex and cerebellum depending on location. Curiously, the left hemisphere of the cerebral cortex presented significantly lower levels of HSP 90 in the animal groups exposed to one or two radiofrequencies. The decrease of this constitutive protein of the nervous cells [21, 22] might indicate a degree of neuronal vulnerability [23]. In contrast, the high levels of HSP-70 in the left hemisphere of animals exposed to a single frequency has been described by some authors as neuroprotection [24]. Both proteins were elevated in the left hemisphere of the cerebellum, indicating a balance between anti-apoptotic/apoptotic mechanisms [25, 26]. In spite of the joint and complementary action of both proteins [27], levels of pre-apoptotic proteins increased significantly in animals exposed to the highest RF levels (Group II, 2.45 GHz).

In the results described here, neither HSP reached levels that were significantly different from the control values in the tissues of the cerebral cortex and cerebellum of rats exposed to the interaction of two radiofrequencies (Group III) (See Figures 3 and 4A, 4B, 4C, 4D). Some authors suggest that exposure to a radiofrequency may stimulate cellular stress in mammals by provoking higher expression of HSP and increasing the risk of cancer [28]. However, analysis of the results of diverse *in vivo* or *in vitro* experiments describing changes in protein expression after non-ionizing radiation have since been revised [29]: some were controversial, others could not be replicated. In the analysis of stress protein levels obtained from rat tissue in this experiment, animals exposed to radiation were compared with non-exposed, non-radiated control animals at all times and under identical experimental conditions (immobilized for the same amount of time in the chamber). This allowed for a more reliable analysis of the data obtained.

The HSP-70 chaperone reached the highest and most persistent levels in the cerebral cortex and the cerebellum 24 hours after radiation (for two hours) in animals exposed to a single frequency and the highest SAR. This cytoprotective protein acts to preserve and restore cellular proteins that are under stress [30] from stimuli such as hyperthermia [31], ischemia [32, 33] and/or oxidative stress [34]. HSP-70 values are regulated by circulating corticoids [35], but there is no relation with glucocorticoid levels or the nature of the acute stimulus [18]. Tissue sensitivity to stress is determined by glucocorticoid receptors (GR), which recent research indicates are jointly regulated through coordinated action of HSP 90 and 70 [36]. This corroborates recent findings in our laboratory, in which GR marking in rat thymocytes after identical exposure in a GTEM chamber at 2.45 GHz increased with increasing SAR and time after exposure [37].

In previous work, we observed that the acute interaction of several radiofrequencies with non-thermal SAR did not increase cellular apoptosis in eight different types of tissue [13]. However, in the current work we found a significant increase in pre-apoptotic activity (see Results). This was described by other authors after *in vitro* radiation of PC12 cell lines [38, 11] or after exposure *in vivo* for one or two months [39]. However, the over-expression of HSP-70 in the cerebral cortex of animals exposed to 900 or 2450 MHz RF attenuated caspase-3 [10] and inhibited neuronal apoptosis [40], thereby diminishing cerebral ischemia in the astrocytes [41] and maintaining the mitochondrial physiology during induced stress through glucose deprivation in the astrocytes [42].

The Specific Absorption Rate (SAR), or proportion of energy deposited per unit of mass in body tissues is the biological index used to determine biological effects and basic restrictions in relation to human exposure to radiation [43, 44]. In this work, the SAR was calculated by the FDTD numerical simulation method. Due to program limitations, the SARs of each RF were averaged to obtain the SAR for both frequencies [45], which only approximates reality. In the experimental radiation model studied and analyzed here, no linear relation was found between electromagnetic exposure and biological effects, since it did not follow a linear dose-response relationship. Several of our own experiments and work by other researchers [46, 47, 9] confirm that greater tissue absorption of energy (SAR) or EMFs does not necessarily induce a greater biological response. Some authors consider that SAR should not be used as a predictor of biological effects or as a quantitative dosimetry property for describing non-thermal effects [48].

Our concluding hypothesis, based on the biological results of this experiment, is that additive biological effects do not exist when two combined signals of different frequencies act simultaneously. As we mentioned earlier (Results, Section 1) our first supporting argument builds on the experimental visualization of the resulting signal with an oscilloscope in the laboratory, using lower frequencies than those applied in the experiment. The combined signal acquired a completely different morphology than either of the individual RFs (see the simulation of the combined signal, Figure 2A), adopting a form similar to a modulated wave. This could locate the debate around the proposal of an action mechanism for the combined signal that is similar to pulse-modulated radiofrequency [49], which some authors associate with specific biological effects of non-ionizing radiation in the human nervous system. Modulated RF signals provoke alterations in electroencephalograms corresponding to sleep [50], wakefulness [51, 52], cerebral flow [53], memory performance tests [54] in humans or convulsive experimental models [55] in the hematoencephalic barrier [56] of rats. Nonetheless, many authors do not accept that the specificity of those non-thermal biological effects is linked to radiation with modulated waves [57].

Legislation for the European Union, including Spain (Regulation establishing protection conditions for public domain radioelectricity, 2001), considers that in situations of simultaneous exposure to different frequencies, the possibility of additive effects of exposure should be taken into account for public protection. However, there is no evidence of a multiple interaction mechanism, and therefore of the effectiveness of those limits [13].

To conclude, in light of the experimental results of this work we propose the following with regard to the interaction of two radiofrequencies, 900 and 2450 MHz, in the nervous tissue:

This interaction provokes energy absorption in the nervous tissue resulting from the combined signals, but does not appear to be the sum of both SARs.

The biological effect of cellular stress in the cerebral cortex and/or cerebellum is related more to the nature of the signal than any additive action of the two combined frequencies, which suggests the possibility of a different action mechanism when multiple signals act on the tissue.

The sub-thermal effects of the combined two-frequency signal constitute a non-linear study biosystem in which a linear cause-effect relationship does not exist.

## METHODOLOGY

### Animals

Forty adult male Sprague-Dawley rats weighing approximately 200 g were used in this study. They were housed in individual cages, with free access to food and water, in an environment maintained at 22±C and subjected to a 12:12 h light/dark regime. All experiments were carried out in accordance with European regulations on animal protection (Directive 86/609), the Declaration of Helsinki and/or the Guide for the Care and Use of Laboratory Animals [58, 59]. All experimental protocols were approved by the Institutional Animal Care and Use Committee of the University of Santiago de Compostela.

### Experimental setup and SAR calculations

#### The experimental radiation system

The experimental rat (R) was immobilized in a methacrylate holder and placed in the region of maximum field uniformity inside the Gigahertz Transverse Electromagnetic (GTEM) chamber [60]. The animal was then exposed to radiation for one or two hours. The experimental system used for applying radiation to the rats is described in López-Furelos et al, [13] and a small explanatory diagram is provided here in Figure 1. Direct Current enables measurement of incident power values (P_IN_) by the PM and of reflected power (P_REF_) by the SA, making it possible to determine the power transmitted (P_TR_) to the GTEM chamber as P_TR_ = P_IN_-P_REF._

#### SAR Calculations

The specific absorption rate (SAR) values were estimated with the aid of SEM-CAD X finite-difference time-domain (FDTD)-based software [45]. A Sprague-Dawley numerical (voxel) phantom rat was used [45], weighing 198.3 g and composed of 60 different tissues assembled into slices 1.15mm thick. Tissue morphologies were obtained by magnetic resonance imaging. The phantom rat was radiated by a plane wave impinging on its left side, with the magnetic field H parallel to its main axis [45].

The electric field value in the simulation was measured experimentally for each case, using an isotropic probe located in the center of the area where the rat would be exposed.

The value of SAR_S_ in each case was estimated by scaling the SEMCAD [45] numerical model rat, taking into account the weight differences among the rats in the three experimental groups of radiated animals. In this way, we have made a uniform scaling (multiplying all the original dimensions of the numerical phantom rat with the same factor), according to the proportionality constant needed to effectively scale the model rat to the model weight [61].

Since the animals were exposed laterally in the GTEM chamber, the left side of the body (as seen from the door of the chamber) experienced a greater field than the right side. This is due to the fact that the electric field within the GTEM increases as the height of the septum decreases. For this reason, and to verify the data obtained in the biological study described here, we simulated the exposure of the numerical model rat (198.3g) at different field levels on both sides, which were experimentally determined in the chamber with an IP in the position occupied by the two sides of the animal cerebrum. Once recorded and introduced into the simulation, a zone study was done for both sides of the cerebrum using the SEMCAD FDTD simulation program. Tables V and VI show the results of this comparison, showing that the SAR on the left side of the cerebrum and cerebellum was always higher than on the right side.

**Tables V and VI:** SAR values in cerebrum and cerebellum and body of a Sprague-Dawley numerical phantom rat, calculated from power (P_TR_), electric field (E_m_) on both sides of the cerebrum (left and right)

	Side	E_m_ [V/m]	Mean SAR of cerebrum [W/kg]	Mean SAR of cerebellum [W/kg]
GROUP I f = 900 MHz P_TR_ = 2 W	Left	50.63	0.0556236	0.0410845
	Right	50.06	0.0402864	0.0250631
GROUP II f = 2450 MHz P_TR_ = 2 W	Left	53.32	0.0492298	0.0907025
	Right	41.30	0.0260181	0.0858929
GROUP I f = 900 + 2450 MHz P_TR_ = 1 + 1 W	Left	43.27	0.03361385	0.05669851
	Right	39.60	0.0266726	0.03580835

**Table:** 

	Side	E_m_ [V/m]	Mean SAR of cerebrum [W/kg]	Mean SAR of cerebellum [W/kg]
GROUP I f = 900 MHz P_TR_ = 4 W	Left	71.42	0.1119	0.0818
	Right	70.99	0.0802	0.0504
GROUP II f = 2450 MHz P_TR_ = 4 W	Left	74.67	0.0965	0.1832
	Right	58.69	0.0596	0.1666
GROUP I f = 900 + 2450 MHz PTR = 2 + 2 W	Left	57.42	0.0628	0.1086
	Right	55.58	0.0495	0.0647

### Experimental design

A total of 40 rats, divided into four groups of 10, were used in the study. The animals in three groups were individually exposed to radiation in the GTEM chamber:

Group I: Irradiation at 900MHz (power = 2 W or 4W).

Group II: Irradiation at 2450MHz (power = 2 W or 4W).

Group III: Simultaneous irradiation at 900MHz (power = 1W or 2W) and 2450MHz (power = 1 W or 2W).

Group IV: The negative control group of 10 rats that were individually not irradiated (negative controls).

Two temporally separate experiments were done (all animal tissues were radiated and processed at different times) as follows:

Experiment 1: The three experimental groups (I-III) were exposed in the GTEM chamber for one hour at maximum power of 2W.

Experiment 2: The three experimental groups (I-III) were exposed in the GTEM chamber for two hours at maximum power of 4W.

All rats in all groups were immobilized in the holder for 1 or 2 hours, during which time the rats in Groups I-III were individually irradiated. Immobilized Group IV animals were placed in the GTEM chamber but were not irradiated. Rats in Groups I-IV were slaughtered 24 h after removal from the GTEM chamber.

### Stress levels indicated by changes in post-exposure rectal temperatures

Rectal temperatures were measured in order to evaluate how radiation, a stimulus that may trigger thermal stress, affected hypothalamic adjustments to the thermoregulatory mechanisms [62]. Temperature was measured with a digital thermometer (Eutech instruments) immediately before placing the animal in the radiation chamber and immediately after exposure (0m).

### Tissue extraction and preparation of cell extracts

After exposure to radiation, the rats were kept alive for a specific amount of time and then given a lethal dose of Pentothal. Tissues from the cerebral cortex and cerebellum were then dissected out under a stereomicroscope (Nikon Eclipse CFI60). The tissue samples were placed in 500 μl of phosphate buffer (PBS; 0.015M phosphate buffer, 0.15M NaCl, pH 7.2) containing 0.1mM pepstatin A, 0.02mM N-(trans-epoxysuccinyl)-L-leucine-4 guanidinobutylamide (E-64), 1mM phenylmethanesulfonyl fluoride (PMSF) and 2mM ethylenediaminetetraacetic acid (EDTA) protease inhibitors (all from Sigma-Aldrich). The samples were disaggregated and homogenized in a Polytron tissue homogenizer (Kinematica AG, Littau, Luzern, Switzerland) at 35,000 rpm for 5 min on ice, and finally ultrasonically lysed in a Branson W-250 sonifier (Branson Ultrasonic Corporation, USA) by means of 5 10-pulse sonication cycles with a 50% duty cycle output. The whole process was performed on ice. The lysate obtained was centrifuged at 15,000 g for 10 minutes at 4°C. The supernatant was then aliquoted and frozen at −80°C until use.

#### Determination of protein concentration

Protein concentration in the tissue extracts was determined by the Bradford method with a Bio-Rad Protein Assay kit (BioRad Laboratories, Germany), using BSA (Sigma-Aldrich, Spain) as standard.

#### Chemiluminiscent enzyme-linked Immunosorbent Assay (ChELISA)

An ChELISA test was applied to detect HSP90, HSP70 and caspase-3. One gram aliquots of protein extract in 100 μl of carbonate-bicarbonate buffer (pH 9.6) were placed in the 96 wells of the ELISA plates (Greiner bio-one, wells of high binding) and incubated overnight at 4oC. The plates were then washed three times with TBS (50mM Tris, 0.15M NaCl, pH 7.4), blocked for 1 h with TBS containing 0.2% Tween 20 (TBS-T1) and 5% non-fat dry milk, incubated for 2 h at 37°C with 100 μl of a 1:100 dilution (in TBS-T1 containing 1% non-fat dry milk) of a murine polyclonal anti-Hsp90, anti-Hsp70 and anti-caspase-3 antibody (Santa Cruz Biotechnology, USA) and washed five times with TBS containing 0.05% Tween 20. For detection of mouse immunoglobulins (Ig), 100 μl of the polyclonal antibody peroxidase-conjugated rabbit anti-mouse Ig (Dako) was diluted 1:1000 in TBS-T1 and incubated for 1 h at 37oC. The wells were washed five times in TBS, then treated with 100 μl of enhanced luminol-based chemiluminiscent substrate for the detection of horseradish peroxidase (Pierce ECL Werten Blotting substrate, Thermo Scientific). After 3 minutes' incubation at 37°C, the luminescence of the plates was read in a fluorometer / luminometer (FLx800, Biotek), and the results were expressed in relative light units (RLU).

### Quantification and statistical analysis

The results shown in the text and figures are expressed as means ±SEM; significant differences (*p* < *0.05*).

a) Two-way ANOVA was used to evaluate stress levels in relation to temperature (Table III and IV). The factors considered were interaction of one or more frequencies and temperature changes before and after radiation.

b) The ChELISA results (Figure 3 and 4) to detect HSP90, HSP70 and caspase-3 in the cerebral cortex and cerebellum, as well as polyclonal antibody were determined by two-way ANOVA, using protein radiation concentration in the right and left hemispheres and the interaction of one or more frequencies. The Holm-Sidak test for multiple comparisons was subsequently applied.

Natural logarithm transformations were applied to the data as needed to obtain normality and homoscedasticity.
